# Prevalence and predictors of medication non-adherence among patients with multimorbidity: A systematic review protocol

**DOI:** 10.12688/hrbopenres.12961.2

**Published:** 2021-03-17

**Authors:** Louise Foley, James Larkin, Richard Lombard-Vance, Andrew W. Murphy, Gerard J. Molloy

**Affiliations:** 1 School of Psychology, National University of Ireland, Galway, Galway, Ireland; 2 HRB Centre for Primary Care Research, Royal College of Surgeons in Ireland, Dublin, Ireland; 3 Discipline of General Practice, National University of Ireland, Galway, Galway, Ireland; 4 HRB Primary Care Clinical Trials Network Ireland, National University of Ireland, Galway, Galway, Ireland

**Keywords:** multimorbidity, medication adherence, study protocol, systematic review

## Abstract

**Introduction:** Patients with multimorbidity are expected to adhere to complex medication regimens in order to manage their multiple chronic conditions. It has been reported the likelihood of adherence decreases as patients are prescribed more medications. Much medication adherence research to date is dominated by a single-disease focus, which is at odds with the rising prevalence of multimorbidity and may artificially underestimate the complexity of managing chronic illness. This review aims to describe the prevalence of medication non-adherence among patients with multimorbidity, and to identify potential predictors of non-adherence in this population.

**Methods:** A systematic review will be conducted and reported according to PRISMA guidelines. PubMed, EMBASE, CINAHL and PsycINFO will be searched using a predefined search strategy from 2009–2019. Quantitative studies will be considered eligible for review if prevalence of medication non-adherence among adults with two or more chronic conditions is reported. Studies will be included in the review if available in English full text. Titles and abstracts will be screened by single review, with 20% of screening cross-checked by a second reviewer. Full-text articles will be screened by two independent reviewers, noting reasons for exclusions. Data extraction will be performed using a predefined extraction form. Quality and risk of bias assessment will be conducted using criteria for observational studies outlined by Sanderson et al. (2007). A narrative synthesis and, if feasible, meta-analysis will be conducted.

**Discussion:** By exploring medication non-adherence from a multimorbidity perspective, the review aims to inform an evidence base for intervention development which accounts for the rising prevalence of patients with multiple chronic conditions.

**Study registration:** The systematic review is prospectively registered in PROSPERO ( CRD42019133849); registered on 12 June 2019.

## Introduction

Multimorbidity has been defined as the presence of two or more chronic conditions in one individual ( Fortin *et al.*, 2012). The rising prevalence of multimorbidity can be attributed to improvements in healthcare and the ageing population associated with the epidemiologic transition ( Omran, 2005). A 2012 UK study involving over 1 million patients reported that 23.2% had two or more chronic conditions, while this rate increased to 65% when the population considered was restricted to those aged 65–84 years ( Barnett *et al.*, 2012). Patients with multimorbidity are placed at increased risk of experiencing fragmented care due to the disease-centric care model currently dominating medical research, education, and practice ( Tinetti *et al.*, 2012). Accordingly, synthesising the relevant evidence existing to date is required to guide future research and practice in the context of multimorbidity.

Adherence refers to the extent to which a person’s behaviours correspond with agreed recommendations from their healthcare provider ( Haynes *et al.*, 2005). As well as changes to lifestyle behaviours, patients with chronic diseases are often expected to adhere to complex drug regimens ( Noël *et al.*, 2007). In the context of multiple conditions, the likelihood of medication non-adherence increases as patients are prescribed more medications ( Benner *et al.*, 2009), with associated risks to health outcomes ( DiMatteo *et al.*, 2002). Such non-adherence poses potential problems for both patients and health systems, highlighting a need to further investigate the occurrence of medication non-adherence and associated factors in multimorbidity according to existing evidence.

Despite knowledge of the rising prevalence of multimorbidity, much intervention development to enhance medication adherence and reviews of the adherence literature are centred on single-disease populations ( Williams *et al.*, 2008). Such a focus is at odds with the rising prevalence of multimorbidity and may lead to an artificial underestimation of the complexity of self-management in chronic disease. One existing systematic review has evaluated medication adherence in older adults with polypharmacy, a phenomenon closely associated with multimorbidity ( Zelko *et al.*, 2016). They cite caregiver burden, impaired hearing, poor cognition and greater number of drugs as predictors of non-adherence in that population ( Zelko *et al.*, 2016). Nevertheless, it has been noted that while relative risk of multimorbidity increases with age, the absolute prevalence of multimorbidity is higher among adults aged under 65 years ( Barnett *et al.*, 2012). Additionally, while multimorbidity and polypharmacy have been branded as “two sides of the same coin” ( Sinnott & Bradley, 2015), it has also been noted that the phenomena may be independent of one another depending on the definition and measurement used in individual studies ( Nicholson *et al.*, 2019). Therefore, a review of the literature inclusive of all adults with multimorbidity is considered necessary to provide the breadth of understanding required to inform intervention development relevant to the whole population of patients with multimorbidity.

Quantitative studies have reported prevalence rates and predictors of medication non-adherence in adult patients with two or more chronic conditions, however to our knowledge no synthesis of this evidence exists to date. Understanding non-adherence to prescribed medications among patients with multiple chronic conditions will provide insight which goes beyond the single-disease focus currently dominating adherence research. The proposed systematic review aims to identify an evidence base to inform research and practice involving patients with multimorbidity, who comprise a large proportion of the population living with chronic disease.

## Research questions

The primary objective of the review is to systematically examine existing evidence relating to prevalence of medication non-adherence and predictors of medication non-adherence among patients with multimorbidity.

The review will specifically address three research questions:

1. What is the prevalence of medication non-adherence among patients with multimorbidity?
2. What are the clinical and psychosocial predictors of medication non-adherence among patients with multimorbidity?
3. Is the method of medication adherence measurement a moderator of non-adherence estimates in multimorbidity?

## Methods

### Study registration

The study is prospectively registered in PROSPERO, the International Prospective Register of Systematic Reviews ( CRD42019133849).

### Study design

The study will be conducted according to the Preferred Reporting Items for Systematic Reviews and Meta-analyses (PRISMA) ( Moher *et al.*, 2009). Each stage of the study selection process will be reported, from title and abstract screening through to selection of full-text articles for inclusion in the review. Reasons for exclusion during full-text review will be reported. A PRISMA flow diagram will be generated to outline each stage of this process. The present systematic review protocol is reported according to PRISMA-P guidelines ( Moher *et al.*, 2015) (see *Reporting guidelines*; Foley, 2019). Any amendments to the study protocol will be documented here and in the associated PROSPERO document.

### Ethics and dissemination

As the study protocol is for a systematic review of existing evidence, ethical approval is not required. The results of the systematic review will be reported in a peer-reviewed journal, presented at relevant health research conferences, and included in a PhD thesis.

## Systematic reviewing

### Types of studies

Experimental and non-experimental quantitative studies reporting prevalence of medication non-adherence in people with two or more chronic conditions will be included in the review. Observational studies – including longitudinal and cross-sectional studies – are anticipated to be the most pertinent study type reviewed. Where intervention studies (randomised and non-randomised controlled trials) report prevalence and/or determinants of medication non-adherence, only baseline data will be extracted and used in the review. The presence of multimorbidity (two or more chronic conditions) must be explicit (i.e. part of study aims and/or inclusion criteria) for studies to be included in the review.

### Population

Adults aged 18 years or older with two or more chronic conditions.

### Outcome

Prevalence of medication non-adherence measured using any relevant method, e.g. self-report, pharmacy data, physical tests, etc.

### Information sources

The review will employ the following electronic databases: PsycINFO, PubMed, EMBASE, and CINAHL. Articles considered eligible for inclusion will be available in English and in full-text from January 2009 to April 2019. The search strategy will combine terms relating to adherence and multimorbidity (see *Extended data*; Foley, 2019). The date range is deemed appropriate considering the scope of adherence prevalence studies conducted in people with two or more chronic conditions since Fortin & colleagues (2012) called for “a more uniform methodology” in multimorbidity research, promoting consistency in how multimorbidity is defined within the literature.

## Data collection, data extraction, study assessment

### Study selection

Studies identified from database searching will be exported to Endnote X8® where duplicated references will be identified and excluded using the ‘find duplicates’ function, and then screened manually for outstanding duplicates by listing studies in order of title. Remaining studies will be exported to Covidence review management software for screening. Titles and abstracts will be screened by a single reviewer (LF will screen 50% and RLV will screen 50%). To assess agreement between reviewers, 20% of these records will be cross-checked by the other reviewer (LF, RLV). Full-text articles will be independently screened by two members of the review panel (LF, JL). Reference lists of all included studies will be independently searched by two reviewers for additional relevant articles. Where disagreement arises between reviewers at any stage, a third reviewer (GJM and/or AWM depending on subject expertise required) will be consulted.

### Data extraction

Data extraction will be performed by two independent reviewers (LF, JL). A pre-defined data-extraction form will be used (see *Extended data*; Foley, 2019) to extract the following: country of publication, citation, study aims, study design, study setting, chronic conditions studied, sample size, participant age, participant gender, definition of multimorbidity used (if applicable), definition of medication adherence, measure of medication adherence, prevalence of medication non-adherence (or non-adherence score), predictors of medication non-adherence (if reported), authors’ conclusions. Where disagreement occurs, a third reviewer (GJM) will be consulted. Where reported data are deemed unclear or insufficient, corresponding authors will be contacted by LF for clarification.

### Quality appraisal

Study-level quality appraisal will be conducted for all included studies by two reviewers (LF, JL) using criteria for assessing quality and risk of bias in observational studies ( Sanderson *et al.*, 2007). Specifically, criteria relate to appropriateness of source population, inclusion/exclusion criteria, measurement methods, methods to deal with design-specific sources of bias, design and/or analytical methods, use of statistics, and declarations of conflict of interest and/or funding sources. While RCT designs may be included in the proposed systematic review, the observational nature of the aforementioned tool is considered appropriate as only baseline observations will be extracted from RCT studies for review. No studies will be excluded on the basis of quality appraisal.

## Data synthesis

### Narrative data synthesis

A narrative synthesis of all reviewed studies will be conducted.

### Assessment of heterogeneity

The I ^2^ statistic will assess heterogeneity, using an alpha level of 0.05 for statistical significance. An I ^2^ value between 50% and 75% indicates high heterogeneity between studies ( Higgins & Thompson, 2002).

### Quantitative data synthesis

Where data support quantitative synthesis, a meta-analysis will be conducted using the metaprop function in R ( R Core Team, 2019). Study-specific estimates will be pooled to estimate the prevalence of medication non-adherence. A random effects model will be employed to account for between-study heterogeneity ( Higgins & Thompson, 2002). To account for asymmetry, the Clopper Pearson method will be used to calculate binomial proportion confidence intervals ( Clopper & Pearson, 1934). The effect of each individual study on the overall estimates of non-adherence prevalence will be assessed using sensitivity analyses by serial exclusion.

### Analysis of subgroups

Where data are sufficient, the following *a priori* moderator analyses will be performed:

1. For method of medication adherence measurement used (e.g. self-report vs. prescription refill vs. serum assay, etc.).
2. For definition of multimorbidity used (e.g. 2 or more chronic conditions vs. 3 or more chronic conditions, etc.).

### Assessment of reporting biases

Publication bias will be assessed by producing and inspecting a funnel plot ( Egger *et al.*, 1997) and by conducting an Egger test for statistical significance ( Sterne *et al.*, 2005).

## Current study status

The systematic review protocol was finalised in March 2019 and the database search was conducted in April 2019. Full-text screening was completed in October 2019. It is anticipated the review will be completed in January 2020.

## Discussion

The review will describe the cumulative evidence relating to medication non-adherence prevalence and relevant predictors among patients with multimorbidity. Understanding non-adherence prevalence and associated factors in this population will reflect the current reality of a rising incidence of complex patients. It is expected the prevalence of medication non-adherence will increase in accordance with the complexity of multimorbidity. A potential limitation relates to the restricted date range for database searching and the use of single-review to screen titles and abstracts. Another potential limitation relates to the heterogeneity associated with the experience of multimorbidity. Accordingly, between-study heterogeneity may not support the conduct of quantitative meta-analysis.

Results of the systematic review will be published in a peer-reviewed journal and disseminated at a range of health research conferences. The systematic review is part of a larger PhD project which aims to identify and understand predictors of non-adherence in multimorbidity in order to guide intervention development to support medication adherence for patients with multimorbidity.

## Data availability

### Underlying data

No underlying data are associated with this article.

### Extended data

Open Science Framework: Prevalence and predictors of medication non-adherence among patients with multimorbidity: A systematic review and meta-analysis. https://doi.org/10.17605/OSF.IO/9Y3RH ( Foley, 2019).

The following files are available as extended data on Open Science Framework (OSF):

- Search Strategy PsycINFO.pdf (search strategy to be used)
- Data Extraction Form.pdf

### Reporting guidelines

Open Science Framework: PRISMA-P checklist for ‘Prevalence and predictors of medication non-adherence among patients with multimorbidity: A systematic review and meta-analysis’. https://doi.org/10.17605/OSF.IO/9Y3RH ( Foley, 2019).

Data are available under the terms of the Creative Commons Zero “No rights reserved” data waiver (CC0 1.0 Public domain dedication).

## References

[ref-1] Barnett K Mercer SW Norbury M: Epidemiology of multimorbidity and implications for health care, research, and medical education: a cross-sectional study. *Lancet.* 2012;380(9836):37–43. 10.1016/S0140-6736(12)60240-2 22579043

[ref-2] Benner JS Chapman RH Petrilla AA: Association between prescription burden and medication adherence in patients initiating antihypertensive and lipid-lowering therapy. *Am J Health Syst Pharm.* 2009;66(16):1471–1477. 10.2146/ajhp080238 19667004

[ref-3] Clopper CJ Pearson ES: The use of confidence or fiducial limits illustrated in the case of the binomial. *Biometrika.* 1934;26(4):404–413. 10.2307/2331986

[ref-21] DiMatteo MR Giordani PJ Lepper HS: Patient adherence and medical treatment outcomes: a meta-analysis. *Med Care.* 2002;40(9):794–811. 10.1097/00005650-200209000-00009 12218770

[ref-4] Egger M Smith GD Schneider M: Bias in meta-analysis detected by a simple, graphical test. *BMJ.* 1997;315(7109):629–634. 10.1136/bmj.315.7109.629 9310563 PMC2127453

[ref-5] Foley L: Prevalence and predictors of medication non-adherence among patients with multimorbidity: A systematic review and meta-analysis. *OSF.* 2019. 10.17605/OSF.IO/9Y3RH PMC7140769 32296748

[ref-6] Fortin M Stewart M Poitras ME: A systematic review of prevalence studies on multimorbidity: toward a more uniform methodology. *Ann Fam Med.* 2012;10(2):142–151. 10.1370/afm.1337 22412006 PMC3315131

[ref-7] Haynes RB Yao X Degani A: Interventions to enhance medication adherence. [update of Cochrane Database Syst Rev]. *Cochrane Database Syst Rev.* 2005; (4): CD000011. 10.1002/14651858.CD000011.pub2 16235271

[ref-8] Higgins JPT Thompson SG: Quantifying heterogeneity in a meta-analysis. *Stat Med.* 2002;21(11):1539–1558. 10.1002/sim.1186 12111919

[ref-9] Moher D Liberati A Tetzlaff J: Preferred reporting items for systematic reviews and meta-analyses: the PRISMA statement. *Ann Intern Med.* 2009;151(4):264–269, W64. 10.7326/0003-4819-151-4-200908180-00135 19622511

[ref-10] Moher D Shamseer L Clarke M: Preferred reporting items for systematic review and meta-analysis protocols (PRISMA-P) 2015 statement. *Syst Rev.* 2015;4(1):1. 10.1186/2046-4053-4-1 25554246 PMC4320440

[ref-11] Nicholson K Makovski TT Griffith LE: Multimorbidity and comorbidity revisited: refining the concepts for international health research. *J Clin Epidemiol.* 2019;105:142–146. 10.1016/j.jclinepi.2018.09.008 30253215

[ref-12] Noël PH Parchman ML Williams JW Jr: The challenges of multimorbidity from the patient perspective. *J Gen Intern Med.* 2007;22(3):419–424. 10.1007/s11606-007-0308-z 18026811 PMC2150619

[ref-13] Omran AR: The epidemiologic transition: a theory of the epidemiology of population change. 1971. *Milbank Q.* 2005;83(4):731–757. 10.1111/j.1468-0009.2005.00398.x 16279965 PMC2690264

[ref-14] R Core Team: A language and environment for statistical computing. Vienna, Austria: R Foundation for Statistical Computing; 2012.2019. Reference Source

[ref-15] Sanderson S Tatt ID Higgins JPT: Tools for assessing quality and susceptibility to bias in observational studies in epidemiology: a systematic review and annotated bibliography. *Int J Epidemiol.* 2007;36(3):666–676. 10.1093/ije/dym018 17470488

[ref-16] Sinnott C Bradley CP: Multimorbidity or polypharmacy: two sides of the same coin? *J Comorb.* 2015;5:29–31. 10.15256/joc.2015.5.51 29090158 PMC5636041

[ref-17] Sterne JAC Becker BJ Egger M: The funnel plot. In HR Rothstein, AJ Sutton, & M Borenstein (Eds.), *Publication bias in meta-analysis: Prevention, assessment and adjustments*. Chichester, England; Hoboken, NJ: Wiley.2005;75–98. 10.1002/0470870168.ch5

[ref-18] Tinetti ME Fried TR Boyd CM: Designing health care for the most common chronic condition--multimorbidity. *JAMA.* 2012;307(23):2493–2494. 10.1001/jama.2012.5265 22797447 PMC4083627

[ref-19] Williams A Manias E Walker R: Interventions to improve medication adherence in people with multiple chronic conditions: a systematic review. *J Adv Nurs.* 2008;63(2):132–143. 10.1111/j.1365-2648.2008.04656.x 18537843

[ref-20] Zelko E Klemenc-Ketis Z Tusek-Bunc K: Medication adherence in elderly with polypharmacy living at home: a systematic review of existing studies. *Mater Sociomed.* 2016;28(2):129–32. 10.5455/msm.2016.28.129-132 27147920 PMC4851507

