# Supplementary material for: Prevalence and predictors of medication non-adherence among patients with multimorbidity: A systematic review protocol
Source: HRB Open Res. 2021 Mar 17;2:29. Originally published 2019 Nov 11. [Version 2] doi: 10.12688/hrbopenres.12961.2 (PMC7140769; doi:10.12688/hrbopenres.12961.2)
Supplement: Peer review history

# Peer review: Reviewer response for version 1

**DOI:** 10.21956/hrbopenres.14044.r27134

**Published:** 2020-02-13

**Authors:** Jacqueline A. Pugh

**Affiliations:** 1 Division of General/Hospital Medicine, Department of Medicine, University of Texas Health Science Center at San Antonio, San Antonio, TX, USA; 2 South Texas Veterans Health Care System, San Antonio, TX, USA

I have reviewed both the protocol paper and the posting at OSF. I am reviewing the protocol after the authors believe they will have finished the data extraction. 

 Overall I do believe this fills a narrow gap in the literature - what is the prevalence of medication non-adherence in all ages of patients with 2 or more chronic diseases. (Not sure if it will include the pediatric literature since not explicitly addressed.) The breadth of the potential diseases that could be included in this review is quite large, as is the type of medication involved. While I concur that previous narrow focus on single disease states or on elderly only has not been adequate, the proposed review seems to somewhat naively view looking at 2 or more diseases as helping us understand non-adherence better. The predictors of non-adherence to be collected do not include type of medication (oral (qd, bid ,tid, etc.), injectable (IM, subcu; daily, weekly, monthly etc.; prn or scheduled; medication types)). This could be crucial to better understanding non-adherence. Already existent literature has noted frequency of medication dosage and number of drugs being prescribed as predictors of adherence. (There is already literature on polypills as a way to improve adherence in those who are prescribed multiple drugs for cardiovascular disease prevention). Other predictors are also out there - cost of medications and other barriers to obtaining them. 

 The authors have faithfully followed the PRISMA-P recommendations for reporting, their search strategy seems appropriate. One modest concern is that they will not exclude any studies on the basis of quality. I would argue that if the study is a case series and therefore cannot really assess prevalence in the underlying population, it should be excluded. I hope this review will lead the authors to looking next at interventions to improve adherence in this population as I predict the non-adherence rates will be relatively high.

Is the study design appropriate for the research question?

Yes

Is the rationale for, and objectives of, the study clearly described?

Yes

Are sufficient details of the methods provided to allow replication by others?

Yes

Are the datasets clearly presented in a useable and accessible format?

Not applicable

Reviewer Expertise:

Multimorbidity, diabetes epidemiology, health services research.

I confirm that I have read this submission and believe that I have an appropriate level of expertise to confirm that it is of an acceptable scientific standard.

# Peer review: Reviewer response for version 1

**DOI:** 10.21956/hrbopenres.14044.r26918

**Published:** 2019-11-29

**Authors:** Deborah E. Patton

**Affiliations:** 1 School of Pharmacy, Queen's University Belfast, Belfast, UK

This is a very well written protocol of a systematic review that aims to identify the prevalence and predictors of non-adherence in adult patients with two or more chronic conditions (multi-morbidity). It recognises that the number of patients with multi-morbidity (commonly prescribed multiple medications) is rising, yet the focus of much adherence research is still on single diseases or individual medications. The findings from this review will help to address an important gap in the current adherence literature. There is a clear rationale provided for conducting this systematic review. The methods detailed in the protocol are clearly described, appropriate and in line with current guidelines for conducting and reporting systematic reviews and the PRISMA-P checklist has been completed.

 I only have a few minor comments/suggestions, to improve the clarity for readers:

- It would be helpful to provide a definition for chronic conditions that will be adopted in the systematic review (e.g. The WHO defines them as: "health problems that require ongoing management over a period of years or decades").
- Examples of ‘Clinical and psychosocial predictors of medication non-adherence’ would be helpful for readers unfamiliar with these terms.
- Under ‘Types of studies’, it is currently unclear if studies including patients aged under 18 years will be excluded or if studies will be included if the mean age of study participants is 18 years or over? 
- The authors mention that “Quantitative studies have reported prevalence rates and predictors of medication non-adherence in adult patients with two or more chronic conditions…” but no references have been provided here.  

Is the study design appropriate for the research question?

Yes

Is the rationale for, and objectives of, the study clearly described?

Yes

Are sufficient details of the methods provided to allow replication by others?

Yes

Are the datasets clearly presented in a useable and accessible format?

Not applicable

Reviewer Expertise:

Medication adherence; polypharmacy; multi-morbidity; community pharmacy; systematic reviews; intervention development; feasibility and pilot studies; pharmacists.

I confirm that I have read this submission and believe that I have an appropriate level of expertise to confirm that it is of an acceptable scientific standard.
